# The Postural and Body Surface Temperature Response of Leisure Horses to Lunging with Selected Lunging Aids

**DOI:** 10.3390/ani14010022

**Published:** 2023-12-20

**Authors:** Małgorzata Maśko, Urszula Sikorska, Marta Borowska, Łukasz Zdrojkowski, Tomasz Jasiński, Małgorzata Domino

**Affiliations:** 1 Department of Animal Breeding, Institute of Animal Science, Warsaw University of Life Sciences (WULS–SGGW), 02-787 Warsaw, Poland; malgorzata_masko@sggw.edu.pl (M.M.); urszula_sikorska@sggw.edu.pl (U.S.); 2 Institute of Biomedical Engineering, Faculty of Mechanical Engineering, Białystok University of Technology, 15-351 Bialystok, Poland; m.borowska@pb.edu.pl; 3 Department of Large Animal Diseases and Clinic, Institute of Veterinary Medicine, Warsaw University of Life Sciences (WULS–SGGW), 02-787 Warsaw, Poland; tomasz_jasinski@sggw.edu.pl

**Keywords:** geometric morphometrics, infrared thermography, posture, body surface temperature, effort, warm-up, lunge training

## Abstract

**Simple Summary:**

Incorporating lunging into a horse’s daily routine proves valuable for enhancing fitness and physical condition. During lunging, horses may either work with a freely moving head (FMH) or with lunging aids (LAs) designed to assist horses in developing a particular skill or exercise. Evaluating the effectiveness of lunging poses a significant challenge for horse owners, riders, trainers, and veterinarians. This study employs non-contact technologies such as geometric morphometrics and infrared thermography to assess lunging efficiency concerning different head and neck positions. The research aims to determine if changes in a horse’s posture correspond to increased metabolic activity, indicated by body surface temperature. Thirteen horses included in the study were lunged using various aids, including chambon (CH), rubber band (RB), triangle side reins (TRs), and without aids (FMH). Images were taken both in visible light and in infrared. Lunging with FMH resulted in a lifted head and lowered back, TRs and RB caused the opposite, while CH induced no posture changes. Horses that lunged with FMH exhibited lower temperatures over a larger area. In contrast, CH led to moderate temperatures over a smaller region. RB resulted in moderate to high temperatures over a broader surface, while TRs led to higher temperatures over a smaller area. The methods proposed in this study offer a means to evaluate the efficiency of lunging in horses.

**Abstract:**

Incorporating lunging into a horse’s daily routine aims to enhance fitness, physical condition, and specific skills or exercises when using lunging aids (LAs). To assess the effectiveness of lunging, non-contact technologies like geometric morphometrics and infrared thermography can be employed. This study seeks to evaluate lunging efficiency based on the horse’s posture and surface temperature when lunging with different head and neck positions. The study aims to determine if changes in a horse’s posture correspond to increased metabolic activity, as indicated by body surface temperature. Thirteen horses included in the study were lunged with chambon (CH), rubber band (RB), and triangle side reins (TRs) as well as with a freely moving head (FMH). Images were taken in visible light and infrared. Principal Component Analysis (PCA) was used to analyze horse posture changes and a Pixel-Counting Protocol (PCP) was used to quantify surface temperature patterns. The horses’ posture exhibited contrasting changes, reflected by a changing centroid shape (*p* < 0.0001) but not size (*p* > 0.05) when lunged with RB and TRs, but not CH. Different (*p* < 0.0001) surface temperature patterns were observed during lunging. FMH lunging resulted in lower temperatures over a larger surface, CH induced moderate temperatures on a smaller area, RB caused moderate to high temperatures across a broader surface, and TRs led to higher temperatures over a smaller region. The studied lunging cases returned different (*p* < 0.0001) surface temperature patterns. Lunging with FMH returned lower temperatures over a larger surface, CH moderate temperatures on a smaller area, RB moderate to high temperatures across a broader surface, and TRs higher temperatures over a smaller region. The proposed methods can be applied to evaluate the efficiency of lunging in horses.

## 1. Introduction

Riding and lunging are crucial elements of a horse’s daily routine, designed to provide the necessary quantity and quality of exercise [1]. Exercise necessitates efficient cooperation among all the physiological systems [2], making both forms of training essential for enhancing the horse’s fitness, performance, and overall physical condition. During lunging, the horse remains within a fixed distance from the person in the center of the circle. The trainer influences the horse through physical presence or signals, teaching it to move around the circle’s perimeter [3]. The evaluation of the intended objectives of lunging [4] is therefore important both in light of the principles of learning theory [5] and the use of lunging aids (LAs). The welfare risks associated with improper lunging stem from the lack of consensus on identifying optimal arousal thresholds [3] and the potential for excessive use of lunging aids [6,7].

When used appropriately, lunging with a chambon (CH) (a compliant elastic aid running from the girth via a headpiece to the bit rings), rubber bands (RBs) (a compliant elastic aid running from the girth via a bit rings to the headpiece), or triangle side reins (TRs) (a stiff aid running from the girth via a bit rings to the other place of girth) can encourage a horse to adopt a correct outline and develop fitness and strength in the relevant musculature [8,9,10,11,12]. The use of LAs should have the practical purpose of helping or assisting the horse in developing specific skills or exercises [7]. For instance, CH encourages the horse to lower the head and round the back, RB encourages the horse to lift and work over its back, and TRs encourage the horse to work in a more collected frame, increasing engagement of the abdominal musculature and the hind limbs [7,12]. Improper practice of lunging, both with and without lunging aids (LAs), may pose potential risks of injury and compromise the welfare of horses [3,7,13]. Moreover, lunging may expose the horse to the risk of being chased, which is discouraged as it can trigger the innate flight response. Elevated stress levels not only hinder learning but also have adverse effects on motivation [14]. Optimal learning occurs when arousal thresholds are minimally elevated [3]. It is crucial to ensure that lunging is free from chasing, flight response elicitation, and excessive arousal levels, especially as this training method is often the initial context in which horses are formally educated [3].

Horse fitness and the evaluation of lunging efficiency are of interest to various horse owners and trainers, including from horse athletes [15,16,17] and leisure horses [18,19,20,21]. While limited studies have explored the impact of LAs on equestrian performance [7], existing research has predominantly focused on the effects of LAs or training reins on equine kinematics [22,23,24,25,26] and muscle activity [27]. For instance, Álvarez et al. [22] used a motion capture system and ground force measuring system to assess the effect of horses’ head and neck position on treadmill locomotion. Byström et al. [23] used a motion capture system to evaluate the effects of draw reins on limb kinematics. Cottriall et al. [27] used electromyographic (EMG) and speed measurements to measure back muscle activity when lunging with LAs. Walker et al. [26] used a motion capture and measurement unit (IMU) system to determine the effect of a Pessoa training aid on equine kinematics. Simons et al. [24] and Pfau et al. [25] used the IMU system to assess the effects of different specific LAs on back kinematics and movement symmetry [24] and back kinematics during trot in-hand and on lunge-line [25], respectively. Because all of these methods require attaching electrodes (EMG) or expensive sensors (IMU) to the horse’s body, or monitoring movement using multiple cameras (motion caption), further research is needed across the spectrum of the different contactless tools available.

A horse’s fitness is often evaluated through standardized exercise tests or tests with a gradual increase in effort, where parameters like heart rate (HR), heart rate variability (HRV), and blood biomarkers are commonly employed [18,19,20,21,22,23,24,25,26,27,28,29,30,31]. However, these tests involve contact with the horse, either through blood sampling [31,32] or the installation of contact measurement sensors [31], which may affect measurements, especially in excitable or stressed horses [33]. Efforts are being made to develop non-contact technologies like infrared thermography to address this challenge. Recent studies have shown positive correlations between body surface temperature and various fitness indicators, including HR [34,35], HRV [34,35], blood lactate concentration [32], blood creatine kinase activity [36], and effort intensity [37].

Infrared thermography detects radiant energy emitted by objects above absolute zero temperature and calculates the object’s temperature, applicable to both inanimate [38] and living objects, such as a horse’s body surface [39]. As exercise intensity increases, the metabolic heat production influenced by muscle metabolism and blood circulation [40,41] rises, necessitating increased heat loss [39,42]. Soroko et al. used infrared thermography to describe variation in back temperature distribution throughout the training cycle [43], Simon et al. studied temperatures of limbs in treadmill training sessions [44], Martins and Silva compared the eye temperature between lunging and ridden workout [1], and other authors assessed the effect of head and neck position during lunging on surface temperature in the distal limb region [12], head and neck region [35], and multiple regions representing almost the entire surface of the horse’s body [45]. However, in the most recent study, Martins and Silva [1] emphasized the need for more conclusive research on horses’ work on the lunge to better understand its effects on the horse’s body. Therefore, this study proposes geometric morphometrics as a non-contact imaging method to assess the effectiveness and quality of the lunge warm-up in the future.

Geometric morphometrics has recently found application in equine medicine for analyzing horse postures by utilizing coordinates of anatomical landmarks and their spatial locations [46,47,48,49,50,51,52,53]. Unlike methods that focus on specific body parts, geometric morphometrics assesses the entire body’s position in space, providing a less subjective and more accurate measurement of horse posture [49,50,51]. Geometric morphometrics emphasizes the shape of individuals or a selected body part, enabling the description of overall morphology [54] and the identification of subtle variations in posture [46,47,48,49,50]. In equine medicine, geometric morphometrics has been introduced to quantify body conformation [52,55], to characterize behavioral postures [47,49,50], feeding behaviors [51], and postural responses to rehabilitation techniques [53] as well as to assess welfare [49,50].

Recognizing that a neutral horse posture is linked to a neutral head and neck position (HNP) [56,57], and that this neutral HNP is characteristic of horses without any load [35], we hypothesized a coincidence between changes in a horse’s posture and body surface temperature in response to lunging exercises and different HNP scenarios. We considered four HNP scenarios: one being neutral during lunging with a freely moving head (FMH), and three being restricted when using various LAs such as the CH, RB, and TRs. The study aimed to evaluate how a horse’s posture changes following lunging with different LAs and whether this posture change coincides with an increase in local metabolic activity, assessed non-invasively through body surface temperature.

## 2. Materials and Methods

### 2.1. Horses

The study was conducted on thirteen (*n* = 13) horses owned by the Warsaw University of Life Sciences (WULS), comprising eight geldings and five mares from two Polish warmblood breeds, predominantly a Polish Halfbred (PHB) horses (*n* = 8) and a minority of Malopolska (MLP) breed horses (*n* = 5). The study was designed as a prospective, interventional study with blinding image processing and analysis. A power analysis was conducted to determine the sample size, considering the minimum–maximum temperature differences in the lateral surface of the neck before and after lunging with FMH [45].

The inclusion criteria for the study were as follows: (1) age between 6 and 20 years; (2) height at withers between 146 and 170 cm; (3) body condition score (BCS) between 2 and 3 on a 5-point scale; (4) the lack of clinical signs of disease; (5) the lack of lameness on a 6-point scale; (6) no anti-inflammatory drugs were administered locally or generally in a month before imaging; and (7) the leisure usage and experience in lunging with FMH and LAs. The sex, breed, and age of the horses were verified using official identification documents. Height at withers was measured with a zoometric stick on the day of the first clinical examination. BCS was assessed through palpation and a visual assessment on a scale from 1 (poor) to 5 (obese) following a standard protocol [58] on the day of the first clinical examination. Assessment of clinical signs of diseases included a general physical examination based on a standard protocol [59]. For general health evaluation, rectal temperature, heart rate, respiratory rate, mucous membrane color and hydration, capillary refill time, and lymph nodes were examined. Lameness was assessed on a scale of 0 (lameness not perceptible under any circumstances) to 5 (minimal weight bearing by limb in motion and/or at rest or a complete inability to move) using orthopedic examination following the American Association of Equine Practitioners (AAEP) guidelines [60]. Verification of drug administration status was based on the official veterinarian documents of horses. Usage and lunging experiences were confirmed through the official horse-working documents of the Didactic Stable of Horse Breeding Division (DSHBD) at WULS. The horses underwent examination twice by the experienced veterinarian (TJ) in the free days of riding.

All horses were individually housed in stalls within a single stable, where they experienced consistent environmental conditions and the same management system at DSHBD at WULS. The study horses were fed three times daily with oats and hay, tailored to each horse’s requirements. Freshwater was available to the horses ad libitum, and a mineral salt block was provided. Aside from free access to a mineral salt block, the horses did not receive any feed supplements or concentrates other than oats for a period of at least six months before the start of the study. Horses had access to a sandy paddock for a minimum 6 h per day.

### 2.2. Lunging Protocol

**Figure 1:**
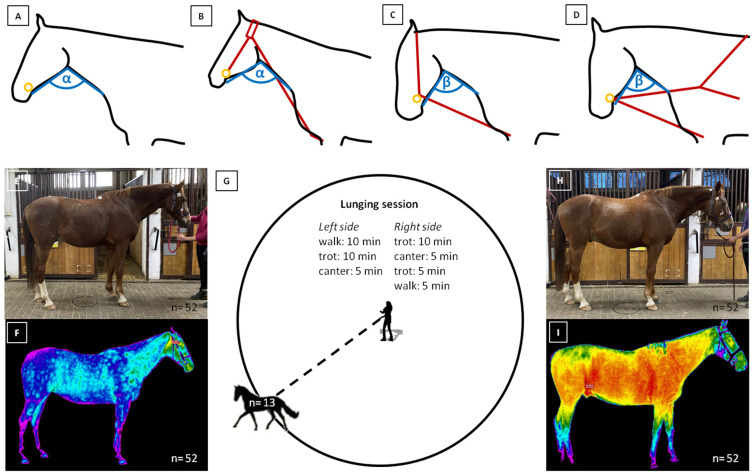
The lunging protocol considering the work with (**A**) freely moving head (FMH), (**B**) chambon (CH), (**C**) rubber band (RB), and (**D**) triangle side reins (TRs). The first imaging was performed before the lunging session in (**E**) visible light and (**F**) infrared. Then, (**G**) horses were lunged. The second imaging was performed after the lunging session in (**H**) visible light and (**I**) infrared. In the subfigures (**A**–**D**), the open head and neck angle (HNA) is marked with α (110°–115°), and the close HNA is marked with β (85°–90°). Moreover, the bit position is marked with an orange ring, and lunging aids (LAs) locations are marked with dark red lines. In the subfigures (**E**,**F**,**H**,**I**), the number of taken images was reported.

All horses followed the same sequence during lunging, according to the procedure: without lunging aids (FMH), with CH, RB, and TRs. The lunging sessions spanned four consecutive days, with each day dedicated to a specific head and neck position. The length of LAs was individually adjusted for each horse to achieve a head/neck angle (HNA) (α; β) of (α) 110°–115° without reins (Figure 1A) or with CH (Figure 1B), as well as (β) 85°–90° with an RB (Figure 1C) or TRs (Figure 1D). The HNA was measured each time during each LA insertion and confirmed through goniometric measurements on visible light images.

The lunging sessions consisted of walk (estimated speed up to 1.5 m/s), trot (estimated speed up to 4.0 m/s), and canter (estimated speed up to 6.0 m/s). The tempo was adjusted individually for each horse. The lunging sessions were conducted as follows: 10 min of walk, 10 min of trot, 5 min of canter, 10 min of trot, 5 min of canter, then 5 min of trot, and finally 5 min of walk. Lunging was performed on both sides of the circle, and session lasted for 50 min (Figure 1G).

All horses were imaged twice, the first time before the lunging session (Figure 1E,F) and the second time directly after the lunging session (Figure 1H,I). Following the second imaging, the horses were walked until they achieved complete rest. Post the lunging session, the horses were not exposed to any additional exercise, ensuring almost a full day of rest between sessions. During their free time, horses were allowed to relax on a sandy paddock.

### 2.3. Horses’ Imaging

All imaging sessions for the horses were conducted indoors in a stable corridor. The corridor, featuring a hard surface, provided a closed and sheltered environment, minimizing the impact of external conditions. The imaging took place over four consecutive days in May, with ambient temperatures ranging from 20.5 to 22.1 °C and humidity ranging from 50.6% to 53.0%. Each imaging session occurred in the morning, starting between 9 AM and 11 AM. The stable corridor was directly connected to the riding hall, ensuring that horses remained indoors between imaging and lunging. Horses were brushed from dirt and mud at least 30 min before imaging and seven self-adherence medical tape markers were applied to one side of their bodies. These markers were placed on this side with less mane. Seven anatomical points chosen for marker placement: (1) the first caudal vertebra (the base of the tail), (2) the lumbosacral joint, (3) the first lumbar vertebra, (4) the intervertebral joint of the 10th thoracic vertebra, (5) the atlantooccipital joint, (6) the temporomandibular joint, and (7) the end of the facial crest. The markers were positioned along the spine and lateral side of the head. Following the second imaging session, the markers were carefully removed without pulling out the hair.

#### 2.3.1. Visible-Light Imaging

All horses were imaged following a previously established protocol for the geometric morphometrics in equids [52,53]. Each horse was led on a halter through the stable corridor by a familiar experimenter (US) and stopped in a spontaneous position. Images were taken on one side of the horse with less mane. A Canon EOS 5D Mk2 digital wide-angle camera (Canon Inc., Tokyo, Japan) was positioned on a tripod 5 m from the horse, with the height adjusted individually to each horse’s height at the withers. The horse stood parallel to the long axis of the corridor, and the central beam of the camera was aligned with the center of the horse’s trunk, parallel to the ground. The camera was positioned at an angle of 90° to the long axis of the corridor. Five images were captured on each occasion, and one image was chosen based on criteria such that all four hooves were positioned on the ground, the head was positioned straight parallel to the long axis of the corridor in a natural position, and no movement artifacts were found. If more than one image was available, one was randomly selected. In total, the same researcher (ŁZ) captured 520 images, and 104 images (52 before lunging and 52 after lunging) were chosen and saved as JPG files.

#### 2.3.2. Infrared Imaging

All horses were imaged following international guidelines for equine thermography [39]. Infrared images were taken on one side of the horse with less mane immediately after taking images in visible light, eliminating the need to move the horse. The digital infrared radiation camera VIGOcam.v50 (emissivity (e) 0.99; VIGOSystem S.A., Ozarow Mazowiecki, Poland) was positioned on a tripod 2 m from the horse at a height individually adjusted to each horse’s height at the withers. The central beam was positioned in the center of the horse’s trunk to be parallel to the ground and to fall at 90° angles onto the horse. Each time, two images were taken, from which one image was selected in which all four hooves were positioned on the ground, the horse’s head was not twisted, and no movement artifacts were found. If more than one image was suitable, one image was randomly selected. A total of 208 images were taken by the same researcher (MM) of which 104 were selected (52 images before lunging and 52 images after lunging). The images were saved as VPR files.

### 2.4. Visible-Light Image Processing

The JPG visible-light images were imported into the tpsUtil software (version 2.31) and a TPS file was built. The tpsDig2 software (version 2.31) was utilized to process the TPS file, and the curve landmarking was manually annotated by the same blinded researcher (MD). Following the methodology outlined by Balcer et al. [53], seven points were initially placed at the marker positions, along with an additional point in the medial canthus of the eye. Subsequently, three points were added between 1st and 2nd points, two points between 2nd and 3rd points, and six points between 3rd and 4th points. Finally, eleven points were added between 4th and 6th points, creating a curve with 30 points that accurately reproduced the shape of the horse’s back from the base of the tail to the end of the facial crest. The coordinates of the curve points were saved in a TPS file.

The TPS file was opened in the tpsDig2 software, and the TPS curve was appended to 30 landmarks (LDs). The LDs from the 1st to 8th were grouped in hindquarter region, the LDs from the 9th to 19th were grouped in back region, and LDs from the 20th to 30th were grouped in head and neck region. The LD coordinates were saved in a TPS file. Subsequently, the TPS file was opened in a notebook, separators were changed from comma to dot, and ID code was added to each image. The ID code contained LAs code (A, FMH; B, CH; C, RB; and D, TRs), time code (0, before lunging; 1, after lunging), and individual code (from 01 to 52).

### 2.5. Visible-Light Images Analysis

The prepared file was imputed to the MorphoJ software (Copyright 2008–2019 Christian Peter Klingenberg, Apache License, Version 2.0, https://morphometrics.uk/MorphoJ_guide/frameset.htm?index.htm, accessed on 20 October 2023). The ID code was used to extract two classifiers: LAs (contained LAs marked by A, B, C, D) and time (contained imaging before and after lunging marked by 0, 1). The database was divided into subsets by lunging classifiers and time classifiers within, as well as by time classifier separately. The Procrustes fit was performed for the whole dataset and each subset, respectively. For the database, the Generalized Procrustes Analysis was performed, Procrutes coordinates were returned in the covariance matrix (CovMatrix), and Principal Component Analysis (PCA) was used. The first three Principal Components (PCs) were displayed on a wireframe graph, and PC scores were visualized on plots where each data point represented one horse. Then, data points were grouped using a classifier LAs or time as a criterion. The confidence ellipses were set using a 0.9 probability.

### 2.6. Infrared Image Processing

The VPR infrared images were imputed to the Therm software (version 2.29.3) (VIGOSystem S.A., Ozarow Mazowiecki, Poland), and the temperature range was set between 28.0 and 38.0 °C (T∈<28;30>) so that the background below the 28.0 °C threshold was marked with black (hexadecimal color code value (HEX) color #000000). Images were saved in BMP file format, with 968 pixels wide × 709 pixels high. The BMP images were imputed to the paint net. (version 4.3.2) software, and the background above the 28.0 °C threshold was manually masked using HEX color #000000, resulting in the non-#000000 pixel area representing the horses’ body surface.

### 2.7. Infrared Images Analysis

The prepared infrared images were imputed to the extcolors package in Python (https://pypi.org/project/extcolors/, accessed on 10 October 2023). The color histogram method was used for color analysis. The images were segmented automatically since the entire image does not constitute the surface of the horse, the surface temperature of which is shades of purple and navy blue for T∈<28;30>, was masked using HEX color #000000. All non-#000000 pixels were counted following Maśko et al. [61]. The pixel colors were defined using HEX color codes from the rgb2hex library (https://colormap.readthedocs.io/en/latest/, accessed on 10 October 2023).

The CIE76 Formula (1):

∆E* = √((∆L*) + (∆a*) + (∆b*))(1)

was used in the CIELAB color space for the calculation of the pixel color. In the CIELAB color space, colors are expressed as three values: L*, a*, and b*. L* is the perceptual lightness. A* and b* are the colors of human vision. For a* and b*, four unique colors, red, green, blue, and yellow, are considered. Thus, the CIELAB color space represents the chrome plane referred to as L*a*b*. In the color histogram method [62] two colors are expressed as:

L_1*, a_1*, b_1*

L_2*, a_2*, b_2*

and the pixel color similarity is estimated as:

∆L* = (L_2* − L_1*)

∆a* = (a_2* − a_1*)

∆b* = (b_2* − b_1*).

The pixel color similarity was used for color grouping and returned the number of pixels belonging to the color. Five color groups corresponding with the temperature scale on the infrared images were considered. Color groups were defined as follows:

Shades of purple and navy blue for T∈<28;30);
Shades of blue for T∈<30;32);
Shades of green for T∈<32;34);
Shades of yellow and orange for T∈<34;36);
Shades of red for T∈<36;38>.

The number of pixels in the given ranges was counted and returned as a percentage of pixels in the range in relation to the total non-#000000 pixels of the horse surface. The results were visualized on a pie chart.

### 2.8. Statistical Analyses

The geometrical data were compared in the MorphoJ software. The effect of classifiers (LAs and time) on the shape and size of the centroid was assessed with the Procrustes ANOVA, with the significance level set as *p* < 0.05. Average observations for all types of LAs and time classifiers were executed and displayed as wireframe graphs.

The numerical data were compared in GraphPad Prism6 software (GraphPad Software Inc., San Diego, CA, USA). Univariate marginal distribution was tested using a Shapiro–Wilk normality test, for demographic data and the percentage of pixels in each range for all subsets (A, 0; B, 0; C, 0; D, 0; A, 1; B, 1; C, 1; D, 1) independently. Since at least one data series in each subset represented non-normally distributed data, the data were presented in box plots using the median and quartiles (lower quartile, upper quartile, minimum, and maximum values). The percentage of pixels in each range was compared between ranges for each subset, as well as between subsets for each range with the Kruskal–Wallis test followed by Dunn’s multiple comparisons test. The significance level was established as *p* < 0.05.

## 3. Results

### 3.1. Descriptive Statistics Results

The included horses had a mean age of 12.62 ± 4.05 years (range: 6 to 18 years) and a height of 160.62 ± 5.32 cm (range: 152 to 168 cm) at the withers. The horses were assessed with a BCS of 3.31 ± 0.48 (range: 3 to 4) on a 5-point scale. All the horses showed no clinical signs of disease and scored zero on a 6-point lameness AAEP scale. None of the horses received anti-inflammatory drugs during the assessment period. The horses were in leisure use for 1 to 2 h a day, for five days a week, including lunging performed in this study. The horses were only used for riding lessons, with riders’ skills ranging from beginner to intermediate levels. No horses were excluded from the study. Specific demographics for included horses are summarized in Table 1.

### 3.2. Postural Response to Lunging

**Figure 2:**
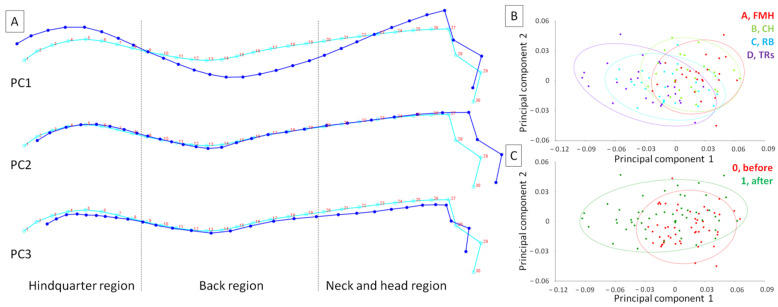
The principal components (PCs) of the horses’ posture as represented by (**A**) the wireframe graph and (**B**,**C**) the scatter plot of PC scores. Light blue curves show the consensus posture of a horse, while dark blue curves show the deformation for PC1, PC2, and PC3. Three regions were separated by dashed lines. The observations were grouped using a classifier as a criterion and marked using confidence ellipses. The observations were grouped for classifier lunging aids (LAs) (A, freely moving head (FMH); B, chambon (CH); C, rubber band (RB); D, triangle side reins (TRs)) and classifier time (0, before lunging; 1, after lunging).

The dataset contained 104 observations, all of which were included for analysis. The total dataset variance was 0.0019, while the variance of the eigenvalues was 0.000000025. Scaling by total variance, the eigenvalue variance was 0.0066, and scaling by both total variance and the number of variables was 0.38. For the first three PCs, PCA returned the following eigenvalues, percentages of variance, and cumulative percentages: PC1: 0.001, 59.54%, 59.54%; PC2: 0.0003, 16.53%, 76.07%; and PC3: 0.0001, 6.18%, 82.25% (Figure 2A). No eigenvalue passed the Kaiser rule (eigenvalues > 1).

One may observe that more horses lunged with FMH represented the PC1-oriented posture with elevated head and hindquarter regions and lowered back region, whereas more horses lunged with TRs represented the PC2-oriented posture with head lowered and stretched out (Figure 2B). Moreover, more horses before lunging represented the PC1-oriented posture, while horses after lunging represented the PC2-oriented posture (Figure 2C).

The classifier LAs and time affected the horse’s posture in terms of centroid shape but not size. The centroid size did not differ between groups of classifier LAs (*p* = 0.277) and time (*p* = 0.054); however, shapes differed between groups of both classifiers (*p* < 0.0001) (Table 2).

Firstly, the database was divided into two subsets annotated by zero for images before lunging and one for images after lunging. Each subset contains 52 observations. For each subset, the effect of classifier LAs was tested. The centroid size did not differ between groups of classifier LAs within both subsets (*p* > 0.05). Shapes did not differ between groups of classifier LAs within both subsets 0 (*p* = 0.062); however, they differed between groups of classifier LAs within subset 1 (*p* < 0.0001) (Table 3).

Secondly, the database was divided into four subsets annotated by A for lunging with FMH, B for lunging with CH, C for lunging with RB, and D for lunging with TRs. Each subset contains 26 observations. For each subset, the effect of classifier time was tested. The centroid size did not differ between groups of classifier time within all considered subsets (*p* > 0.05), whereas shapes differed between groups of classifier time within subset A (FMH, *p* = 0.028), subset C (RB, *p* < 0.0001), and subset D (TRs, *p* < 0.0001) (Table 4).

**Figure 3:**
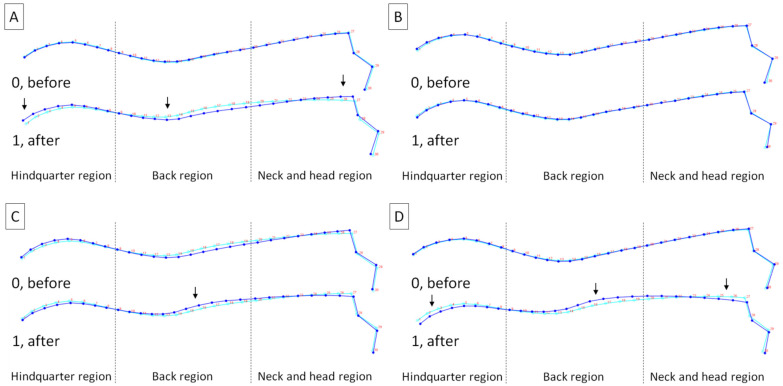
Average observations of the horses’ posture within (**A**) subset A (freely moving head (FMH)), (**B**) subset B (chambon (CH)), (**C**) subset C (rubber band (RB)), and (**D**) subset D (triangle side reins (TRs)) before (0) and after (1) lunging. Light blue curves represent the average dorsal profile of all horses and dark blue curves represent the average dorsal profile for displayed classes. Three regions were separated by dashed lines. Regions with detected differences (reported in Table 3 and Table 4) were marked with arrows.

Regions with detected differences in horses’ posture were marked with arrows on the wireframe graph of average observations (Figure 3). Within subset A, horses after lunging with FMH demonstrated a posture with an elevated atlantooccipital joint in the neck and head region, lowered middle of the back region, and elevated base of the tail in the hindquarter region (Figure 3A). Within subset B, horses did not demonstrate posture changes both before and after lunging with CH (Figure 3B). Within subset C, horses after lunging with RB demonstrated a posture with an elevated the middle of back region (Figure 3C). Within subset D, horses after lunging with TRs demonstrated a posture with a lowered atlantooccipital joint in the neck and head region, elevated the middle of the back region, and lowered base of the tail in the hindquarter region (Figure 3D).

### 3.3. Body Surface Response to Lunging

**Figure 4:**
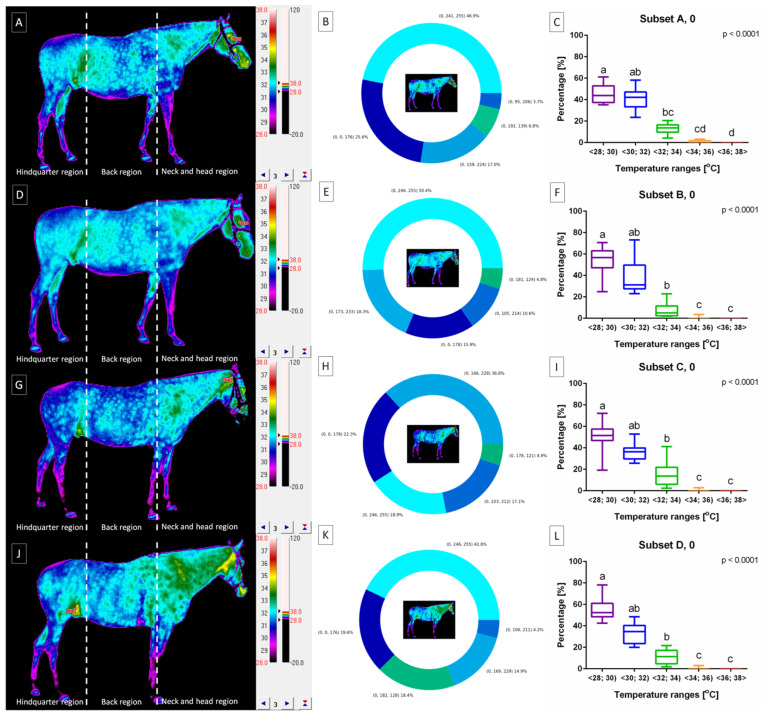
The horse’s body surface profile before lunging with (**A**–**C**) freely moving head (FMH) (subset A, 0), (**D**–**F**) chambon (CH) (subset B, 0), (**G**–**I**) rubber band (RB) (subset C, 0), and (**J**–**L**) triangle side reins (TRs) (subset D, 0). (**A**,**D**,**G**,**J**) infrared images with three regions separated by dashed lines. (**B**,**E**,**H**,**K**) pie charts with visualized percentage of pixels in the ranges. (**C**,**F**,**I**,**L**) comparison of pixel percentage between ranges. Data in box plots are described with lower quartile, median, and upper quartile, with whiskers representing minimal and maximal values. Lowercase letters indicate differences between ranges for *p* < 0.05.

**Figure 5:**
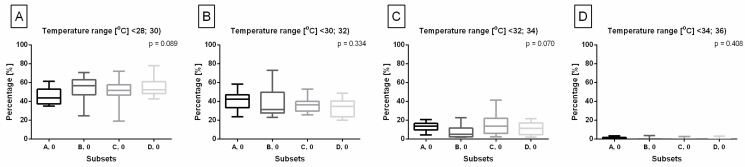
Comparison of pixel percentage of the horse’s body surface profile between subsets before lunging. Pixel percentage in ranges: (**A**) T∈<28;30), (**B**) T∈<30;32), (**C**) T∈<32;34), and (**D**) T∈<34;36) were compared. There were no pixels in range T∈<36;38>. Data in box plots are described with lower quartile, median, and upper quartile, with whiskers representing minimal and maximal values. No differences between subsets were reported for *p* < 0.05.

The horse’s body surface profile before lunging was displayed in Figure 4 for horses lunged with FMH (Figure 4A–C), CH (Figure 4D–F), RB (Figure 4G–I), and TRs (Figure 4J–L) separately. Horses included in all subsets demonstrated body surface profiles with the highest percentage of pixels in ranges T∈<28;30) and T∈<30;32), lower percentage of pixels in range T∈<32;34), and the lowest percentage of pixels in ranges T∈<34;36) and T∈<36;38>. No differences were found between ranges T∈<28;30) and T∈<30;32); between ranges T∈<32;34) and T∈<34;36); as well as between ranges T∈<34;36) and T∈<36;38> (Figure 4C,F,I,L). Moreover, no differences were found between subsets within each range (Figure 5); thus, the horse’s body surface profile before lunging was considered homogeneous.

**Figure 6:**
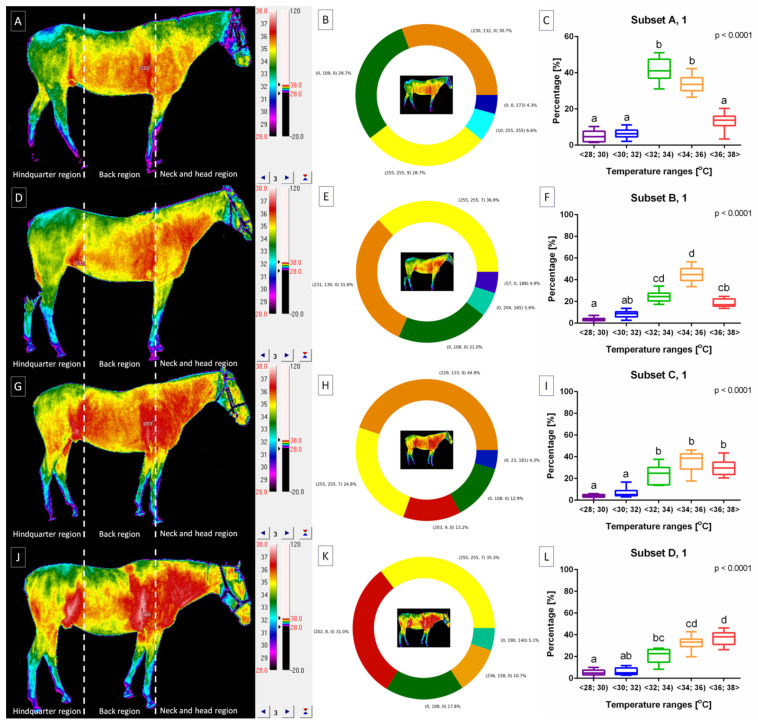
The horse’s body surface profile after lunging with (**A**–**C**) freely moving head (FMH) (subset A, 1), (**D**–**F**) chambon (CH) (subset B, 1), (**G**–**I**) rubber band (RB) (subset C, 1), and (**J**–**L**) triangle side reins (TRs) (subset D, 1). (**A**,**D**,**G**,**J**) infrared images with three regions separated by dashed lines. (**B**,**E**,**H**,**K**) pie charts with visualized percentage of pixels in the ranges. (**C**,**F**,**I**,**L**) comparison of pixel percentage between ranges. Data in box plots are presented with lower quartile, median, and upper quartile, with whiskers representing minimal and maximal values. Lowercase letters indicate differences between ranges for *p* < 0.05.

The horse’s body surface profile after lunging was displayed in Figure 6 for horses lunged with FMH (Figure 6A–C), CH (Figure 6D–F), RB (Figure 6G–I), and TRs (Figure 6J–L) separately. Horses included in subset A demonstrated body surface profile with a higher percentage of pixels in ranges T∈<32;34) and T∈<34;36) as well as lower percentage of pixels in ranges T∈<28;30), T∈<30;32), and T∈<36;38>. For subset A, no differences were found between ranges T∈<28;30), T∈<30;32), and T∈<36;38> as well as between ranges T∈<32;34) and T∈<34;36) (Figure 6C). The horses represented cooler areas in the hindquarter region and warmer areas in the cranial part of the back region as well as the ventral part of the neck and head region, as shown in Figure 6A.

Horses included in subset B demonstrated a body surface profile with the highest percentage of pixels in ranges T∈<32;34) and T∈<34;36), lower percentage of pixels in ranges T∈<30;32) and T∈<36;38>, as well as the lowest percentage of pixels in range T∈<28;30). For subset B, no differences were found between ranges T∈<28;30) and T∈<30;32), T∈<30;32) and T∈<36;38>, T∈<32;34) and T∈<36;38>, as well as T∈<32;34) and T∈<34;36) (Figure 6F). The horses represented cooler areas in the hindquarter region and warmer areas in the cranial part of the back region as well as the ventral part of the neck and head region, as shown in Figure 6D.

Horses included in subset C demonstrated body surface profile with a higher percentage of pixels in ranges T∈<32;34), T∈<34;36), and T∈<36;38> as well as a lower percentage of pixels in ranges T∈<28;30) and T∈<30;32). For subset C, no differences were found between ranges T∈<28;30) and T∈<30;32) as well as between ranges T∈<32;34), T∈<34;36), and T∈<36;38> (Figure 6I). The horses represented cooler areas in the hindquarter region and warmer areas in the whole back region as well as the whole neck and head region, as shown in Figure 6G.

Horses included in subset D demonstrated body surface profile with the highest percentage of pixels in ranges T∈<34;36) and T∈<36;38>, lower percentage of pixels in ranges T∈<30;32) and T∈<32;34), as well as the lowest percentage of pixels in range T∈<28;30). For subset D, no differences were found between ranges T∈<28;30) and T∈<30;32), T∈<30;32) and T∈<32;34>, T∈<32;34) and T∈<34;36>, as well as T∈<34;36) and T∈<36;38) (Figure 6L). The horses represented cooler areas in the hindquarter region and warmer areas in the cranial part of the back region as well as the whole neck and head region, as shown in Figure 6J.

**Figure 7:**
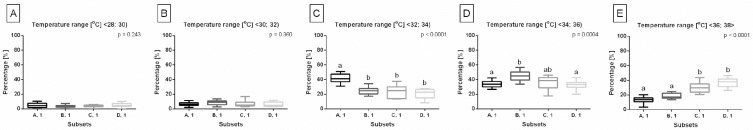
Comparison of pixel percentage of the horse’s body surface profile between subsets after lunging. Pixel percentage in ranges: (**A**) T∈<28;30), (**B**) T∈<30;32), (**C**) T∈<32;34), (**D**) T∈<34;36), and (**E**) T∈<36;38> were compared (after lunging. Data in box plots are presented with lower quartile, median, and upper quartile, with whiskers representing minimal and maximal values. Lowercase letters indicate differences between subsets for *p* < 0.05.

Moreover, no differences were found between subsets within ranges T∈<28;30) (Figure 7A) and T∈<30;32) (Figure 7B). However, within range T∈<32;34), higher percentage of pixels was noted in subset A than subsets B–D (Figure 7C). Within range T∈<34;36), higher percentage of pixels was noted in subset B than subsets A and D. And within range T∈<36;38>, a higher percentage of pixels was noted in subsets C and D than subsets A and B. Thus, the horse’s body surface profile after lunging was considered heterogeneous.

## 4. Discussion

### 4.1. The Most Relevant Results

The growing popularity and interest in less invasive measuring approaches for investigating the training efficiency of sport, race, and leisure horses underscore the importance of considering how horses react to working with LAs and whether this affects training efficiency [63]. Summarizing the most important results, one may observe the distinct changes in horses’ posture during lunging with various head and neck positions. The change in horses’ posture after lunging with FMH included elevation of the neck and head region, lowering of the back region, and elevation of the hindquarter region. This change in horses’ posture was accompanied by a small warming surface in the cranial part of the back region and the ventral part of the neck region. No changes in horses’ posture after lunging with CH were noted; however, a large warming surface at moderate temperature in the cranial part of the back region and the ventral part of the neck region was noted. The change in horses’ posture after lunging with RB included elevation in the back region, which was accompanied by a larger warming surface at moderate and high temperatures in the whole back and neck regions. The change in horses’ posture after lunging with TRs included lowering the neck and head region, elevation of the back region, and lowering the hindquarter region. This change in horses’ posture was accompanied by a large warming surface at high temperatures in the front of the back region and the whole neck region. Consequently, the methodology employed in this study holds practical applications for the broader equine industry.

### 4.2. Postural Response to Lunging

Understanding the potential effect of commonly used LAs on both equine performance and welfare is crucial. This knowledge is essential for selecting the appropriate LAs to meet specific training or rehabilitation goals and to ensure the safe use of the equipment [64,65]. Lunging, a training technique for horses, involves having the horse move in a large circle. It is widely utilized by horse owners, riders, trainers, and veterinarians for various purposes, including communication establishment and muscle strengthening, particularly in the hindquarters [66]. During lunging without any external load, a freely moving horse typically lowers the base of its head and neck, relaxing the muscles, and resulting in an open HNA [67] and a neutral HNP [35].

In contrast, when saddled, the HNA often remains closed due to the horse’s position on the bit, responding to the rider’s rein aids transmitted through the bit [68]. The use of additional equipment, such as LAs, allows for achieving the desired bit position even without a rider [9]. LAs alter the HNP through forces acting on the bit, head, breast, girth, and withers. Specifically, the RB and TRs tend to close the HNP, whereas the CH has the opposite effect of opening it [8]. The CH connects the bit to the girth via the top of the head [45], RB connects the bit to the girth elastically to reduce the HNA [67], while TRs link the bit to the girth in a way that stiffly reduces HNA [8,68]. Both HNP and the varying degrees of elasticity or stiffness in the LAs have an impact on the thoracolumbar kinematics [22] and the movement of the forelimbs and hindlimbs [8], thereby influencing the work of the horse’s muscles and the stability of ligament structures [69]. During lunging with close HNA and position on a bit, the horse typically lifts the thoracolumbar region, engaging the muscles, especially in the hindquarter region, which increases the range of movement of the fore- and hindlimbs [8,22,69]. Therefore, lunging with LAs is a training technique used to enhance balance and engage core muscles, especially in the back and hindquarters regions [66].

In this study, after lunging with FMH, horses straightened their heads when standing, leading to an open HNA, which aligns with previous research findings [35,67]. However, the horses in the study also raised their heads, contradicting the typical behavior of lowering the base of the head and neck while relaxing the muscles, as reported in previous studies [35,67]. It is possible that the horses in these studies were not sufficiently relaxed after lunging with FMH. This aspect warrants further investigation, for example, considering behavioral indicators of relaxation [70]. After lunging with RB and TRs, horses raised their back, leading to a more rounded posture and opening, which aligns with previous research findings [22]. In this case, when lunging with TRs, a lowering of the hindquarters, head, and neck was also observed, a posture that has been regarded as the most desirable in previous studies [8,22,69]. However, the lack of dorsal elevation following lunging with CH requires further investigation. It is worth noting that this study did not account for the kinematic aspects and range of limb movement [8]. However, it is important to recognize that previous research did not employ geometric morphometrics for evaluating horse posture after lunging. To address these gaps in the obtained results and their interpretation, further research in a more comprehensive experimental setup is needed. However, these initial results suggest that the geometric morphometrics method could potentially be effectively incorporated into the assessment of the efficiency and quality of the lunge warm-up.

### 4.3. Body Surface Response to Lunging

The efficiency rate of converting chemical energy to mechanical energy during exercise is 20% [71]. Approximately 80% of the produced energy is emitted as metabolic heat, leading to an increase in surface body temperature [72]. Given that metabolic heat production is influenced by muscle metabolism and blood circulation [40,41,44], and that the HNP may affect blood circulation in the local musculature [35], this study examined the change in surface body temperature globally rather than locally. In recent research, Becker-Birck et al. [35] reported an increase in superficial body temperature after lunging in only three regions of interest (ROIs) on the horse’s neck. In a subsequent study, Maśko et al. [45] demonstrated a similar increase in superficial body temperature in the same three ROIs identified by Becker-Birck et al. [35], as well as in eight additional regions corresponding to subsequent body areas on the chest, back, and rump. This study introduces a novel approach for assessing surface temperature considering the temperature distribution across the entire body surface. It can be evaluated without the need for image segmentation using ROIs [61], minimizing associated inaccuracies. This method offers great convenience by eliminating the requirement for time-consuming segmentation and the subjective determination of ROIs. Such inaccuracies, which might include background fragments, can significantly impact the obtained results. It is hoped that the color-pixel-counting protocol proposed in this study can be applied on a larger scale for further assessments of the effectiveness and quality of lunge warm-up.

In the Becker-Birck et al. [35] study, no differences in the mean body surface temperature of the neck and shoulder regions were observed between lunging in hyperflexion and with an FMH. This suggests that HNP may not affect the thermographically measured emissions of metabolic heat. However, subsequent studies [45], as confirmed in this study, demonstrated such differences. In our study, horses lunged with FMH showed a small warming surface in the cranial part of the back region and the ventral part of the neck region. In comparison to FMH results, horses lunged with the CH displayed a larger warming surface at moderate temperatures in the cranial part of the back region and the ventral part of the neck region. These results are consistent with previous studies [45], where lunging with CH led to higher temperatures in ROIs marked on the back of the neck, the entire neck, and the spine regions compared to lunging with FMH. On the other hand, horses lunged with RB demonstrated a larger warming surface at both moderate and high temperatures in the entire back and neck regions, contradicting previous studies [45] where the temperature in the ROIs marked on the back of the neck and the entire neck regions was lower after lunging with RB compared to FMH. Compared to the results of lunging with an FMH, horses lunged with TRs exhibited the most extensive warming surface at high temperatures in the front of the back region and the entire neck region. These findings align with previous studies [45], indicating that after lunging with TRs, the temperature in ROIs marked on the back of the neck, the entire neck, the spine area, and three ROIs on the hindquarter was higher than after lunging with FMH. These findings suggest that working with all LAs increases the overall metabolic heat emission from a larger part of the horse’s surface compared to lunging with FMH. It can be carefully suggested that the use of TRs not only promotes the activity of superficial muscles but also facilitates the transfer of energy and force between the hindlimbs, hindquarters, and back [73], as indicated by the horse’s body surface profile.

### 4.4. Limitations

Equine LAs are frequently employed to influence the horse’s HNP, thereby impacting spinal kinematics and stride length [9,22,74]. They are also used to enhance the engagement of the horse’s core musculature and hind limbs, aiming to improve propulsion, muscle recruitment, or hypertrophy [12]. However, evaluating kinetic parameters and direct muscle activity often requires contact methods such as EMG [27], IMU [24,25], and a motion caption [22,23,26]. The contact nature of these measurements, the need for a complex measurement setup, and the associated costs of equipment and software limit their practical implementation in everyday stable routines. This study employs cost-effective and straightforward imaging methods that can be easily used by horse owners, trainers, and competitors in any stable setting. While GM and thermographic-based PCP require further validation, these preliminary studies showcase their potential utility in assessing the effectiveness of daily training. Consequently, further research involving a larger cohort of horses is necessary to investigate the influence of variables such as age, sex, breed, training background, and current activities on the outcomes. These factors can significantly impact both lunging behavior and surface temperatures in horses. For instance, optimal arousal levels during lunging are likely to vary among horses, considering sex, breed, and individual experiential differences [3]. Additionally, sex and breed have been shown to influence horses’ effort, recovery during sports performance, and surface temperature patterns [37].

It is worth noting that this preliminary research provides results affected by the research design. The sequence of lunging was structured such that all horses were lunged without aids (FMH) on the first day, with a CH on the second day, RB on the third day, and TRs on the fourth day. The sequence aimed to ensure that the use of LAs did not impact the measurement results after lunging with FMH, and that the severity of FNA changes increased over time. Consequently, the compliant elastic aid supporting an open head and neck position (CH) was implemented first, followed by the compliant elastic aid favoring a closed head and neck position (RB). Finally, the stiff aid supporting a closed head and neck position (TRs) [8,9,10,11,12] was used. While this sequence was chosen for specific reasons, further studies incorporating a different or mixed sequence of LAs are necessary to determine whether the order of aid usage influenced the study outcomes. Additionally, it would be intriguing to conduct similar research with varying training durations, considering the observed significant effect of lunging time on centroid shape. This preliminary study employed a lengthy lunge time (50 min), which aligns with the working time for leisure and school horses [12,45,75]. Given that the recommended duration of effective lunge work for sport horses is shorter (20 min) [3], subsequent studies should explore similar measurements at different intervals, such as after 20, 30, 40, and 50 min of lunging.

Another limitation of this study is the absence of specifications regarding the type of exercise and the accumulation of physiological stress. In the current research, the distinction between aerobic and anaerobic exercise cannot be conclusively confirmed, particularly given that leisure horses exhibit hematological indicators of both exercise types during regular use [75]. To definitively categorize the exercise type, blood tests would be required, rendering the previously employed non-invasive protocol impractical. Nevertheless, for future research, expanding the experimental protocol to include blood tests is recommended to assess comprehensive recovery of the exercising muscles, the degree of muscle fatigue, accurate energy supply through feed, and various indicators of complex metabolic activity [37,76]. Despite these considerations, the absence of differences in the horses’ posture and thermal pattern before exercise suggests that physiological stress and fatigue accumulation were either low or that complete recovery was achieved. It is noteworthy that after the lunging session, the horses were not subjected to any additional exercise and spent over half a day on a sandy paddock to facilitate full relaxation. During their free time, horses had the opportunity to stand in close proximity to other horses, engage in social interactions, or consume hay—a preferred means of relaxation after training [77].

By underscoring these limitations, it becomes evident that further research is warranted to ascertain the reliability of the methodology employed in this study for broader applications in equine contexts.

## 5. Conclusions

Geometric morphometrics and the color-pixel-counting protocol enable the visualization of changes in the horses’ posture, potentially influenced by lunging with FMH and various LAs. The horses’ posture exhibited opposing changes after lunging with FMH compared to lunging with RB and TRs, with lunging using CH showing no discernible effect on the final posture. Each head and neck position during lunging resulted in effective heating of the body surface, ranging from low temperatures after lunging with FMH to moderate temperatures on a smaller body surface after lunging with CH, to moderate and high temperatures on a larger body surface after lunging with RB, and finally to high temperatures on a smaller body surface after lunging with TRs. Horses responded to lunging with different LAs by detectable and repeatable changes in both posture and body surface temperature in non-contact imaging methods. Therefore, both imaging techniques may be applied to assess the efficiency of horses’ work on the lunge.

## Figures and Tables

**Table 1:** Specific demographics of included horses (sex, breed, age, body condition score (BCS), height, lameness score, training background, and current training activities).

No	Sex	Breed	Age	BCS	Height	Lameness Score	Training Background	Current Training
1	G	PHB	15 years	3	166 cm	0	leisure work for 10 years	1–2 h/day, 5 days/week
2	G	PHB	17 years	4	155 cm	0	leisure work for 13 years	1–2 h/day, 5 days/week
3	M	MLP	18 years	3	167 cm	0	leisure work for 14 years	1–2 h/day, 5 days/week
4	M	PHB	10 years	3	168 cm	0	leisure work for 5 years	1–2 h/day, 5 days/week
5	G	MLP	8 years	4	164 cm	0	leisure work for 4 years	1–2 h/day, 5 days/week
6	G	MLP	12 years	3	166 cm	0	leisure work for 8 years	1–2 h/day, 5 days/week
7	M	MLP	16 years	3	154 cm	0	leisure work for 11 years	1–2 h/day, 5 days/week
8	G	PHB	14 years	4	160 cm	0	leisure work for 9 years	1–2 h/day, 5 days/week
9	M	MLP	10 years	3	161 cm	0	leisure work for 8 years	1–2 h/day, 5 days/week
10	G	PHB	18 years	3	159 cm	0	leisure work for 14 years	1–2 h/day, 5 days/week
11	G	PHB	6 years	4	152 cm	0	leisure work for 2 years	1–2 h/day, 5 days/week
12	M	PHB	12 years	3	160 cm	0	leisure work for 8 years	1–2 h/day, 5 days/week
13	G	PHB	8 years	3	156 cm	0	leisure work for 2 years	1–2 h/day, 5 days/week

G, gelding, M, mare, Polish halfbred (PHB) horse (*n* = 8) and the rest a Malopolska (MLP) breed.

**Table 2:** The effect of classifiers (LAs, time) on the horses’ posture (centroid size and shape).

Centroid Size	SS	MS	df	F	*p*
LAs	59,554.18	19,851.39	3	3.17	0.277
Time	26,833.46	26,833.46	1	4.15	0.054
**Shape**	**SS**	**MS**	**df**	**F**	***p***
LAs	0.051	0.0003	168	11.29	**<0.0001**
Time	0.017	0.0003	56	9.78	**<0.0001**

Sum of squares (SS); mean squares (MS); significance level (*p* < 0.05); the significant effects of the classifier were marked with bold font in the *p*-value column.

**Table 3:** The effect of classifier lunging aids (LAs) on the horses’ posture (centroid size and shape) within subsets 0 (before lunging) and 1 (after lunging).

Centroid Size	SS	MS	df	F	*p*
subset 0, before	2971.08	990.36	3	0.22	0.880
subset 1, after	126,982.85	42,327.62	3	6.44	0.090
**Shape**	**SS**	**MS**	**df**	**F**	***p***
subset 0, before	0.009	0.00005	168	1.36	0.062
subset 1, after	0.06	0.0004	168	18.66	**<0.0001**

Sum of squares (SS); mean squares (MS); significance level (*p* < 0.05); the significant effects of the classifier were marked with bold font in the *p*-value column.

**Table 4:** The effect of classifier time on the horses’ posture (centroid size and shape) within subsets A (lunging with freely moving head (FMH)), B (lunging with chambon (CH)), C (lunging with rubber band (RB)), and D (lunging with triangle side reins (TRs)).

Centroid Size	SS	MS	df	F	*p*
subset A, FMH	12,871.57	12,871.57	1	2.16	0.155
subset B, CH	29,150.35	29,150.35	1	5.29	0.304
subset C, RB	1486.08	1486.08	1	0.36	0.553
subset D, TRs	53,725.23	53,725.23	1	8.29	0.083
**Shape**	**SS**	**MS**	**df**	**F**	***p***
subset A, FMH	0.002	0.00003	56	1.40	**0.028**
subset B, CH	0.001	0.00002	56	0.93	0.626
subset C, RB	0.01	0.0002	56	11.0	**<0.0001**
subset D, TRs	0.02	0.0004	56	21.1	**<0.0001**

Sum of squares (SS); mean squares (MS); significance level (*p* < 0.05); the significant effects of the classifier were marked with bold font in the *p*-value column.

## Data Availability

The data presented in this study are available on request from the corresponding author. The data are not publicly available due to the privacy policy of stable and horse owners.
